# Obituary

**Published:** 1907-05-15

**Authors:** 


### OBITUARY.

THEODORE D. BUHL: President of Parke, Davis and Co., of Detroit, dropped dead on the street in New York City, from heart failure April 7th. The following expression of the feelings of those interested in the great work of this large corporation should be of interest to the dental profession.

Ten and a half years ago Theodore D. Buhl cast in his lot with Parke, Davis & Co. Throughout that period he has given it the benefit of his large experience, his sound judgment, his great power in the commercial world, his granite credit reared on an unwavering honesty. As President of the house he was the perfect type of integrity and fidelity to all the stockholders. His high sense of duty as a trustee pledged to administer the property and guard the interests of others, was ever uppermost in his thoughts. The peculiar responsibilities and hazards of the work and obligations as purveyors to the Medical profession and to suffering humanity, were to him always a solemn appeal. The ultimate triumph of character in business was with him a conviction as deep and strong as instinct. The remote future and the distant prize concerned him more than the present gain.

As a director Mr. Buhl was the soul of courtesy, kindness and deference. As an employer he was considerate, thoughtful, mindful of the comfort, interests and claims of his employes. To their grievances he gave always a patient and attentive ear. He encouraged the manly expression of honest opinion, and when it differed from his own his effort was to convince and pursuade, not to invoke his authority or impose his will.

As stockholders, employes and executives of Parke, Davis & Company we record this testimony to the lasting service rendered by our lamented President. To the members of the bereaved family we offer our warm and heartfelt sympathy. May strength be theirs to bear their sorrow. May they find much comfort in the memory of a life rich in well-doing and in good repute.

